# Cardiovascular Characteristics and Outcomes of Young Patients with COVID-19

**DOI:** 10.3390/jcdd8120165

**Published:** 2021-11-26

**Authors:** Antonin Trimaille, Sophie Ribeyrolles, Charles Fauvel, Corentin Chaumont, Orianne Weizman, Thibaut Pommier, Joffrey Cellier, Laura Geneste, Vassili Panagides, Wassima Marsou, Antoine Deney, Sabir Attou, Thomas Delmotte, Pascale Chemaly, Clément Karsenty, Gauthier Giordano, Alexandre Gautier, Pierre Guilleminot, Audrey Sagnard, Julie Pastier, Baptiste Duceau, Willy Sutter, Victor Waldmann, Théo Pezel, Delphine Mika, Ariel Cohen, Guillaume Bonnet, the Critical COVID-19 France Investigators

**Affiliations:** 1 Centre Hospitalier Régional Universitaire de Strasbourg, 67000 Strasbourg, France; antonin.trimaille@chru-strasbourg.fr; 2 Institut Mutualiste Montsouris, 75014 Paris, France; sophie.ribeyrolles@gmail.com; 3 Centre Hospitalier Universitaire de Rouen, 76000 Rouen, France; charles_fauvel@orange.fr (C.F.); corentinchaumont@hotmail.com (C.C.); 4 Centre Hospitalier Régional Universitaire de Nancy, 54511 Vandœuvre-Lès-Nancy, France; Orianne.weizman@gmail.com (O.W.); gauthier.giordano@gmail.com (G.G.); 5 Centre Hospitalier Universitaire de Dijon, 21000 Dijon, France; thibaut.pommier@hotmail.fr (T.P.); guilleminot.pierre@gmail.com (P.G.); audreysagnard@gmail.com (A.S.); july.bonzay@hotmail.fr (J.P.); 6 Hôpital Européen Georges Pompidou, Université de Paris, 75015 Paris, France; cellier.joffrey@gmail.com (J.C.); willy.sutter@gmail.com (W.S.); victor.waldmann@gmail.com (V.W.); 7 Centre Hospitalier Universitaire d’Amiens-Picardie, 80000 Amiens, France; laura.geneste@hotmail.fr; 8 Centre Hospitalier Universitaire de Marseille, 13005 Marseille, France; vassili.panagides@gmail.com; 9 Faculté de Médecine et de Maïeutique, GCS-Groupement des Hôpitaux de l’Institut Catholique de Lille, Université Catholique de Lille, 59800 Lille, France; marsou.w@gmail.com; 10 Centre Hospitalier Universitaire de Toulouse, 31400 Toulouse, France; antoine.deney@gmail.com (A.D.); clement.karsenty@hotmail.fr (C.K.); 11 Centre Hospitalier Universitaire de Caen-Normandie, 14000 Caen, France; sabir.attou@gmail.com; 12 Centre Hospitalier Universitaire de Reims, 51100 Reims, France; tdelmotte@chu-reims.fr; 13 Institut Cardiovasculaire Paris Sud, Massy, 91300 Paris, France; pascale.chemaly@laposte.net (P.C.); gautier.alx@gmail.com (A.G.); 14 Paris Cardiovascular Research Center (PARCC), Paris Translational Research Center for Organ Transplantation, INSERM, UMR-S970, Université de Paris, 75015 Paris, France; bduceau@gmail.com (B.D.); unbonnet@gmail.com (G.B.); 15 Hôpital Lariboisière, APHP, Université de Paris, 75010 Paris, France; theo.pezelccf@gmail.com; 16 Signaling and Cardiovascular Pathophysiology, Inserm, UMR-S 1180, Université Paris-Saclay, Chatenay-Malabry, 92296 Paris, France; delphine.mika@u-psud.fr; 17 Hôpital Saint Antoine, 75012 Paris, France; 18 Unité Médico-Chirurgicale de Valvulopathies et Cardiomyopathies, Université de Bordeaux, Hopital Cardiologique Haut-Lévêque, 33600 Pessac, France

**Keywords:** COVID-19, SARS-CoV-2, young, myocarditis, pericarditis

## Abstract

Although 18–45-year-old (y-o) patients represent a significant proportion of patients hospitalized for COVID-19, data concerning the young population remain scarce. The Critical COVID France (CCF) study was an observational study including consecutive patients hospitalized for COVID-19 in 24 centers between 26 February and 20 April 2020. The primary composite outcome included transfer to the intensive care unit (ICU) or in-hospital death. Secondary outcomes were cardiovascular (CV) complications. Among 2868 patients, 321 (11.2%) patients were in the 18–45-y-o range. In comparison with older patients, young patients were more likely to have class 2 obesity and less likely to have hypertension, diabetes and dyslipidemia. The primary outcome occurred less frequently in 18–45-y-o patients in comparison with patients > 45 years old (y/o) (16.8% vs. 30.7%, *p* < 0.001). The 18–45-y-o patients presented with pericarditis (2.2% vs. 0.5%, *p* = 0.003) and myocarditis (2.5% vs. 0.6%, *p* = 0.002) more frequently than patients >45 y/o. Acute heart failure occurred less frequently in 18–45-y-o patients (0.9% vs. 7.2%, *p* < 0.001), while thrombotic complications were similar in young and older patients. Whereas both transfer to the ICU and in-hospital death occurred less frequently in young patients, COVID-19 seemed to have a particular CV impact in this population.

## 1. Introduction

Since the onset of the coronavirus disease 2019 (COVID-19) pandemic, a common notion is that it would mainly concern the elderly while sparing young adults. For this reason, preventive measures have been primarily focused on the elderly [1]. However, it rapidly appeared that COVID-19 could affect patients of any age. At the beginning of the pandemic in South Korea, where testing was also available for asymptomatic patients, more than half of confirmed cases were under 50 years old (y/o) [2]. In addition, in a report of the first COVID-19 cases analyzed by age in the United States, 20% of 508 hospitalized patients were 20–44 y/o [3]. Among 63,103 patients hospitalized for COVID-19 in the United States, 3222 (5.0%) were 18–34 y/o [4]. Severe COVID-19 does not spare young people, who account for nearly 15% of the 1591 patients admitted to the intensive care unit (ICU) in an Italian cohort [5]. Finally, the decision to initially reserve vaccination for the elderly caused a shift of the epidemic towards the younger generations [6].

The main features and risk factors for severe forms of COVID-19 have been identified in several case series [5,7,8]. Among these, old age and cardiovascular (CV) risk factors emerged as strong predictors of worse outcomes [4,9,10,11,12]. In addition, the impact of severe acute respiratory syndrome coronavirus 2 (SARS-CoV-2) on the CV system is common and polymorphic, including cardiac injury, arterial and venous thrombotic events, acute heart failure, myocarditis, and pericarditis [13].

Detailed data concerning the young population remain sparse, especially concerning the CV implications of COVID-19. Through a multicenter cohort of COVID-19 hospitalized patients, we aimed to determine characteristics and outcomes of young patients with a description of CV complications.

## 2. Materials and Methods

### 2.1. Study Design

The Critical COVID-19 France (CCF) study is a retrospective, observational, multicenter study including consecutive adult patients hospitalized for COVID-19 between 26 February and 20 April 2020, in 24 French centers (NCT04344327) [14,15]. Briefly, a confirmed case of SARS-CoV-2 infection was defined as a positive result on real-time reverse transcriptase polymerase chain reaction (RT-PCR) of nasopharyngeal swab. A probable case was identified by typical chest computed tomography (CT) patterns in the event of inconclusive or unperformed laboratory tests. Patients hospitalized in general wards and emergency departments were included in the study. Patients directly admitted to the ICU were excluded. Patients < 18 y/o were excluded. Patient baseline information included demographic characteristics, co-existing medical conditions, and medications. Clinical parameters and biological findings were recorded at admission. Alcohol consumption was not systematically reported due to its difficult retrospective assessment, complex association with cardiovascular risk and unclear correlation with COVID-19. All medical interventions, including anticoagulation treatment and complementary testing, were left to the discretion of the referring medical team. The details of the anticoagulation regimen according to age are provided in Supplementary Table S1.

Young patients were defined as 18–45-year-old (y-o) patients at admission, which is a common threshold to designate young adults in the CV literature [16] and CV risk assessment [17]. We performed an additional sensitivity analysis by dividing the study population into four groups of ten years (18–30 y/o, 31–40 y/o, 41–50 y/o, and >50 y/o), to illustrate the exact age where COVID-19 mortality and severity significantly increase.

The CCF study was declared to and authorized by the French Data Protection Institute (Commission Nationale Informatique et Liberté, CNIL, reference 2207326v0) and was conducted in accordance with the 1975 Declaration of Helsinki. No extramural funding was used to support this work. The authors are solely responsible for the design and conduct of this study, all study analyses and the drafting of the paper, and its final content. A specific service independent of the authors was used for editing the paper.

### 2.2. Outcomes

The primary composite outcome was ICU transfer or in-hospital death. Secondary outcomes were CV complications during COVID-19 diagnosed by the referring medical team with all available data. Acute coronary syndrome was defined as evidence of myocardial ischemia based on the association of symptoms (mainly chest pain), electrocardiographic changes (ST segment modification, new left bundle branch block, new pathological Q waves), troponin elevation, ischemic imaging evidence (wall systematized motion abnormality on echocardiography when performed) and identification of a coronary thrombus by angiography [18]. Only one of these signs, especially an isolated troponin elevation, was not sufficient to diagnose acute coronary syndrome. Acute heart failure was confirmed in the presence of typical symptoms and signs (i.e., dyspnea, pulmonary crackles, peripheral edema, elevated jugular venous pressure) of congestive heart failure with echocardiographic confirmation of structural or functional cardiac abnormalities [19]. Acute pulmonary embolism (APE) was diagnosed by computed tomography pulmonary angiogram (CTPA) performed using a multidetector scanner after intravenous injection of high concentration iodinated contrast agent [14]. Acute pericarditis was defined by the presence of at least two of the four following criteria: pericarditic chest pain, pericardial rubs, new widespread ST-elevation or PR depression on electrocardiogram and/or pericardial effusion on transthoracic echocardiography [20]. Acute myocarditis was diagnosed as a panel of clinical arguments (chest pain, new onset heart failure, electrocardiographic changes, troponin elevation, exclusion of coronary artery disease) associated with compatible imaging, including functional and structural abnormalities on transthoracic echocardiography and, when feasible, cardiac magnetic resonance (CMR) imaging (i.e., in centers equipped with dedicated SARS-CoV-2 CMR imaging) [21]. Since the differential diagnosis between acute myocarditis and acute coronary syndrome can be difficult, the final diagnosis applied in every doubtful case was based on the CMR diagnosis. Ischemic stroke was suspected in front of typical neurological symptoms and confirmed by brain imaging (CT or MR imaging) analyzed by an expert team [22].

### 2.3. Statistical Analysis

This work was prepared in compliance with the STROBE checklist for observational studies (Supplemental material). Categorical data were reported as counts and percentages. Continuous data were reported as mean (±standard deviation) for normally distributed data and as median (interquartile range) for non-normally distributed data. Comparisons used the chi-squared test or Fisher’s exact test for categorical variables and Student’s t test or the Mann–Whitney–Wilcoxon test, as appropriate, for continuous variables. Univariate and multivariate logistic regression were used to identify risk factors for occurrence of the primary outcome in young patients. Sensitivity analyses were conducted according to the sex in young patients, by treating the age as an ordered class factor and in population without comorbidity. As a prospective cohort study on a disease not well known at the time, no sample size calculation was carried out.

A two-tailed *p* < 0.05 was considered statistically significant. All data were analyzed using R software, version 3.6.3 (R Project for Statistical Computing).

## 3. Results

### 3.1. Baseline Characteristics

**Figure 1:**
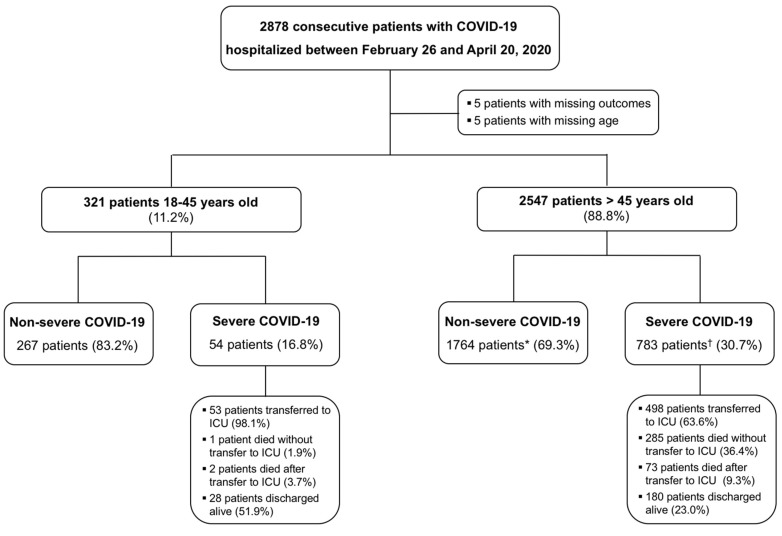
Flowchart of the study. * Three patients with missing final status (0.2%). ^†^ Four patients with missing final status (0.2%). Abbreviation: COVID-19, coronavirus disease 2019; ICU, intensive care unit.

Among 2878 patients hospitalized for COVID-19 in 24 centers between 26 February and 20 April 2020, 2868 were analyzed after exclusion of five (0.2%) with missing outcomes and five (0.2%) with missing age. Among them, 321 (11.2%) patients were 18–45 y/o (Figure 1). Baseline characteristics according to age are presented in Table 1. Compared with older patients, young patients were more frequently obese (36.0% vs. 29.6%, *p* = 0.034), with a higher mean body mass index (BMI) mainly driven by a higher proportion of patients with BMI > 35 kg/m^2^ (18.0% vs. 10.1%, *p* = 0.001) (Table 1). Young patients suffered less frequently from hypertension (9.2% vs. 56.1%, *p* < 0.001), diabetes (6.3% vs. 25.9%, *p* < 0.001) and dyslipidemia (4.7% vs. 30.9%, *p* < 0.001). In addition, young patients had less comorbidities and less medications before hospitalization compared to older patients. Anticoagulation regimens during hospitalization according to the age group are reported in the Supplementary Table S1.

### 3.2. Clinical, Laboratory and Radiological Data on Admission

At admission, there was no difference concerning NYHA functional class distribution between young and older patients (44.6% vs. 52.1% in class III or IV, *p* = 0.087) (Supplementary Table S2). Signs of congestive heart failure were evidenced in 2.2% of young patients and 7.3% of older patients (*p* = 0.001).

Laboratory data at admission are shown in Supplementary Table S2. Respiratory (partial pressure of oxygen), inflammatory (C-reactive protein, lymphocytes) and coagulation parameters (platelets, activated partial thromboplastin time ratio, prothrombin rate, fibrinogen, D-dimers) were significantly less altered in young patients compared to those in older patients (for all *p* < 0.05). The proportion of patients with B-type natriuretic peptide (BNP) or N-terminal-pro-brain natriuretic peptide (NT-pro-BNP) elevation (11.9% vs. 57.0%, *p* < 0.001) and troponin elevation (15.1% vs. 24.4%, *p* < 0.001) was significantly lower in young patients (Supplementary Figure S1).

There was no significant difference between young and older patients concerning COVID-19 severity assessed by chest CT.

We also analyzed baseline characteristics of young patients according to sex. There was no significant difference in the distribution of cardiovascular risk factors between men and women. Interestingly, men had a higher CRP level than women (82.5 mg/L vs. 56.7 mg/L respectively, *p* = 0.002).

### 3.3. Outcomes

In the overall population, the primary outcome occurred in 837 (29.2%) patients: 551 (19.2%) patients were transferred to the ICU and 286 (10.0%) died without transfer to the ICU. In comparison with older patients, the 18–45-y-o population was less likely to experience the primary outcome (16.8% vs. 30.7%, *p* < 0.001), mainly because of a lower in-hospital death rate (0.9% vs. 14.0%, *p* < 0.001) without significant difference concerning ICU transfers (16.5% vs. 19.6%, *p* = 0.22) (Table 2). Mean time between admission and transfer to the ICU was 2.4 ± 3.3 days in young patients and 2.7 ± 3.6 days in older patients (*p* = 0.53). Intubation and mechanical ventilation were required in 31 (9.7%) young patients and in 339 (13.3%) older patients (*p* = 0.08). Non-invasive ventilation and high-flow nasal cannula therapy were used in 5 (1.6%) and 11 (3.4%) young patients and in 75 (2.9%) and 142 (5.7%) older patients, respectively (*p* > 0.05 for both).

Acute pericarditis (2.2% vs. 0.5%, *p* = 0.003) and acute myocarditis (2.5% vs. 0.6%, *p* = 0.002) were more frequently diagnosed in young patients in comparison with older patients (Supplementary Figure S2). Detailed electrocardiographic and echocardiographic data of patients with myocarditis and pericarditis are provided in the Supplementary Tables S3 and S4. Young patients were less affected by heart failure (0.9% vs. 7.2%, *p* < 0.001). Two patients with myocarditis were also included in the acute heart failure definition. The occurrence of acute coronary syndrome (0.3% vs. 1.4%, *p* = 0.17), acute pulmonary embolism (5.0% vs. 3.5%, *p* = 0.25) and ischemic stroke (0.3% vs. 0.8%, *p* = 0.50) did not differ between the young and older patients. Rates of cardiovascular complications stratified by the presence or absence of primary outcome during hospitalization in young patients are shown in the Supplementary Table S5.

Outcomes of young patients according to sex are shown in the Supplementary Table S6. There was a statistical trend for a higher frequency of the primary outcome among men in comparison with women (36 men (20.7%) vs. 18 women (12.2%), *p* = 0.062). Young men experienced longer length of stay than women (7.0 ± 4.7 days vs. 5.8 ± 4.8, *p* = 0.033). Regarding cardiovascular complications, the numbers were too small to analyze statistical difference, except for higher frequency of pulmonary embolism in men (14 (8.1%) vs. 2 (1.4%), *p* = 0.013).

Sensitivity analysis dividing the population into four subgroups by 10-years age groups (18–30 y/o, 31–40 y/o, 41–50 y/o, and >50 y/o) did not reveal any specifically higher-risk age group. The risk of pericarditis and myocarditis appeared to be uniformly distributed within these subpopulations. The threshold of 45 years of age was confirmed by this sensitivity analysis (Supplementary Table S7).

Among the 54 young patients who presented the primary outcome, 13 patients (24%) had a BMI above 35 kg/m^2^. Among these patients with grade 2 obesity or higher (*n* = 50), 13 (26%) presented the primary outcome: 13 (26%) were transferred to the ICU among which one died (2%).

### 3.4. Risk Factors for Severe COVID-19 in Young Patients

In univariate logistic regression analysis, factors associated with the occurrence of the primary outcome in young patients were (Supplementary Table S8): male gender (odds ratio (OR) 1.86, confidence interval (CI) 95% (1.01–3.51), *p* = 0.045), history of hypertension (OR 2.92, CI 95% (1.22–6.64), *p* = 0.017), NYHA functional class III or IV at admission (OR 4.65, CI 95% (2.29–10.10), *p* < 0.001), heart rate (OR 1.05, CI 95% (1.03–1.07), *p* < 0.001), respiratory frequency (OR 1.09, CI 95% (1.05–1.14), *p* < 0.001), temperature (OR 1.74, CI 95% (1.30–2.33), *p* < 0.001), inspired oxygen fraction (FiO_2_) in oxygen support (OR 1.09, CI 95% (1.06–1.13), *p* < 0.001), sepsis-induced coagulopathy (SIC) score ≥ 4 (OR 6.08, CI 95% (2.26–21.70), *p* < 0.001) and quick sequential organ failure assessment (qSOFA) score ≥ 1 (OR 3.78, CI 95% (1.79–8.79), *p* < 0.001). In the multivariate logistic regression analysis, male sex and BMI were independently associated with the occurrence of the primary outcome in young patients, with adjustment for center, age, sex male, BMI, history of hypertension, smoking and previous CV complication (Table 3).

In a sensitivity analysis of the population without any comorbidities (*n* = 665), young patients had a higher BMI (*p* = 0.037) and a better clinical presentation at admission in comparison to older patients, translated by a lower proportion of patients with NYHA functional class III or IV and higher oxygen saturation (*p* < 0.05 for both) (Supplementary Table S9). Respiratory, inflammatory and coagulation parameters were significantly less perturbed in young patients compared to older patients. The proportion of patients with an elevated BNP/NT-pro-BNP level was lower in young patients (*p* < 0.001). Among patients without any comorbidities, the primary outcome occurred significantly less frequently in young patients than in older patients (14.1% vs. 25.8%, *p* = 0.001). Except for myocarditis being more frequently found in young patients (*p* = 0.009), there was no significant difference between young and older patients for CV complications in this population without any comorbidities (*p* > 0.05 for all).

We then analyzed the associations between cardiac biomarkers such as natriuretic peptide and cardiac troponin, and outcomes in the young patients (Supplementary Tables S10 and S11). Of the 185 young patients with available troponin dosage, 28 (15.1%) had increased troponin level. Increased troponin level was associated with the primary outcome (10 patients (35.7%) vs. 27 patients (17.2%), *p* = 0.045) and were more often transferred to the ICU (10 patients (35.7%) vs. 26 patients (16.6%), *p* = 0.036). The small number of young patients with increased BNP/NT-pro-BNP (*n* = 19) did not allow to find any association between this biomarker and outcomes.

We also performed a comparison of patients transferred to the ICU stratified by the transfer timing (Supplementary Table S12). In this analysis, we did not observe any difference between patients transferred to the ICU before the first 24 h of hospitalization and those transferred after 24 h.

## 4. Discussion

Derived from a multicenter observational cohort of 2868 hospitalized patients with COVID-19, our study was designed to describe the determinants and prognosis of young patients with COVID-19. The main results are as follows: (i) young patients account for a significant part of hospitalized patients with COVID-19 (11.2%); (ii) young patients have significantly less comorbidities, especially CV risk factors, except obesity; (iii) no difference was found regarding ICU transfers between young and older patients, even though mortality was lower amongst the younger patients; and (iv) COVID-19 had a particular CV impact in young patients.

### 4.1. Typical Profile of a Young Hospitalized COVID-19 Patient

Among 2868 consecutives COVID-19 patients hospitalized in 24 centers in France, we found that 18–45-y-o patients represented 11.2% of the overall population, which is consistent with previous reports [2,3,4,5].

Several studies have described the main features of hospitalized patients [5,7,23]. A possible association between ethnicity and COVID-19 outcomes has been recently pointed out [24]. Although SARS-CoV-2 seems to be a non-discriminatory virus, infecting both healthy individuals and those with known medical history, nearly half of patients hospitalized for COVID-19 presented with comorbidities at admission, especially CV risk factors [11,12,23]. In a report of 5700 hospitalized patients with COVID-19 in New York, the most frequent comorbidities were hypertension (57%), obesity (42%) and diabetes (34%) [23]. In our study, young patients presented with less comorbidities, in particular less CV risk factors, such as hypertension, diabetes and dyslipidemia, than older patients. Obesity was the only feature to be more frequently observed in young patients (36.0%), with a proportion of those with a BMI > 35 kg/m2 significantly higher in young patients compared with older patients. Obesity has been reported as a risk factor for severe COVID-19 [10,25,26]. In a previous report, morbid obesity was associated with a higher risk of death or need for mechanical ventilation in 18–34-y-o patients [4]. Another study using health records from almost 7 million people in England highlighted that the effect of obesity on the risk of severe COVID-19 was greatest in 20–39-year-olds and decreased after 60 y/o with very little effect over 80 y/o [26]. Many hypotheses could explain this relation. First, obesity is associated with CV diseases, which have been reported as risk factors for severe COVID-19 [13]. Secondly, it is a predisposing factor for hypoventilation syndrome in ventilated patients [25]. Third, obesity may lead to a marked dysregulation of the immunologic response and cytokine profiles within adipose tissue [25]. Finally, previous works have shown high ACE2 expression in adipose tissue, suggesting a mechanism by which excess adiposity may drive greater susceptibility to COVID-19 [27]. Altogether, these data pave the way for an impact of obesity on patients with COVID-19 proportional to its intensity. Young patients may be particularly exposed to this risk factor, given the high proportion of those with a BMI > 35 kg/m^2^.

Hypertension, diabetes and previous coronary artery disease were also associated with worse outcomes [7,13,23]. By multivariate analysis, we only found male gender to be associated with the occurrence of the primary outcome in young patients. By a sensitivity analysis of the population without any comorbidities, we confirmed that the young patients conserved their better clinical and biological profiles at admission in comparison with older patients. In this analysis, the primary outcome occurred more frequently in older patients, reinforcing the hypothesis of an independent worse effect of age.

### 4.2. Outcomes of Young Patients with COVID-19

In some previous studies, age had the strongest impact on prognosis [9,10], and older patients were more likely to experience worse outcomes. In the study of Guan et al. on 1099 patients in China, the mean age of patients who presented with the primary composite outcome of transfer to the ICU, mechanical ventilation or death was significantly higher (63 y/o, range 53–71 y/o) than the one of those without the primary outcome (46 y/o, range 35–57 y/o, *p* < 0.001) [8]. Likewise, we found a lower death rate in young patients, but the proportion of those transferred to the ICU was similar in comparison with older patients. In line with our results, a previous work investigated the clinical profile and outcomes of 3222 18–34 y-o adults hospitalized for COVID-19 in the United States found a relatively low incidence of death but substantial rates of adverse outcomes with 21% who required intensive care and 10% who required mechanical ventilation [4].

Data on the effect of age on cardiovascular complications during COVID-19 are sparse. A previous meta-analysis of observational studies investigated cardiovascular complications in hospitalized COVID-19 patients and the impact of cardiovascular risk factors on mortality [28]. While a significant interaction of age with death was observed, a meta-regression analysis did not identify age as significant predictor of cardiovascular complications. Despite predominant respiratory symptoms during COVID-19, systemic manifestations, such as coagulopathy or cardiac injury, have been described [13]. Many hypotheses have been raised to explain cardiac injury mechanisms [13], such as type 1 or 2 myocardial infarctions, myocarditis, stress-induced cardiomyopathy and cytokine storm. In our study, troponin and BNP/NT-pro-BNP elevations were less important in young patients. The lower BNP/NT-pro-BNP level was in line with a lower rate of acute heart failure in young patients compared with older patients. Compared to older patients, younger patients are less exposed to COVID-19-related myocardial injury, except for myocarditis, due to lower inflammation levels related to less severe sepsis and better respiratory parameters on admission. According to the Fourth Universal Definition of Myocardial Infarction, myocardial injury is defined by an elevated value of troponin ≥ 99 th percentile [29]. While it is a pre-requisite for the diagnosis of myocardial infarction, an isolated troponin elevation was not sufficient to retain this diagnosis as prespecified in the Methods. Thus, the overall lower troponin level in young patients may be explain by the greater predisposition to cardiac injury in older patients.

In our study, while young patients experienced, overall, a lower troponin level, acute myocarditis occurred more frequently in young patients. This paradox might be explained by other causes of troponin elevation, which were more frequently encountered in older patients than in young patients, including acute heart failure, inflammation, renal dysfunction, anemia or hypoxemia. In addition, a higher prevalence of myocarditis in young patients was expected. A predominant incidence of myocarditis in young patients is known, and this population has been identified as a predictor of worse outcomes [30]. In a case-series of 58 patients with H1N1 influenza-related myocarditis during the 2009 pandemic, the mean age was 32 y/o, which was lower than the age of patients with seasonal influenza [31]. In addition, acute pericarditis, a well-known complication of viral infections, was also more frequent in young patients in our study. Like many viruses, SARS-CoV-2 could lead indirectly to pericardial inflammation and effusion secondary to the immunological response [32]. As myocarditis, acute pericarditis is typically encountered in young patients [33]. Further studies are needed to confirm these findings in the COVID-19 context.

In contrast with a higher occurrence of inflammation-related CV complications, young patients experienced less acute heart failure. This observation is probably explained by a lower prevalence of chronic heart failure and CV conditions at admission among young patients. COVID-19-related thrombotic complications such as acute coronary syndrome, ischemic stroke and acute pulmonary embolism were not significantly different between young and older patients. These findings were surprising given that young patients had a better coagulation profile at admission and as a very low incidence of acute coronary syndrome in non-COVID young patients is known in the literature [34,35].

### 4.3. Study Limitations

Our study has several limitations. First, while primary outcome happened less frequently in young patients compared with in older patients, this contrast is mainly driven by a strong difference in the death rate. The proportion of patients transferred to the ICU, reflecting the severe forms of COVID-19, was similar in both young and older patients. These data are not in line with the overall better health of young patients and may suggest that other factors might explain the similar COVID-19 severity. Viral load, assessed by the ΔCt measured in RT-PCR, has emerged as a useful marker of severity [36]. Our work was not designed to assess viral load, and we cannot exclude a difference in this parameter between young and older patients. In addition, this observation may also reflect that those physicians in the wake of the pandemic were more prompt to admit young patients to the ICU with a higher survival probability than that in older patients. Second, the retrospective nature of the data collection should be emphasized. However, the short time between the patient’s hospitalization and data recording (median (interquartile range) = 14 [9–19] days) allowed to easily recover a large amount of interest variables. Third, our study potentially lacks power in the analysis of factors associated with the primary outcome and of CV complications. The rate of some CV complications is low, although this is the largest report in the literature so far. Larger population sizes may reveal differences in acute coronary syndrome, acute pulmonary embolism and stroke incidences between young and older patients. In addition, the CCF study excluded patients directly hospitalized to the ICU, while patients transferred to the ICU after a quick passage in a triage unit were included. We cannot exclude that young patients directly admitted to the ICU have a different profiles and specific outcomes. However, the analysis of patients transferred to the ICU according to the transfer timing, did not show any difference in term of age, cardiovascular risk factors or outcomes. Fourth, there was no systematic testing for cardiac complications. However, since all diagnosis were retrospectively recorded, even in patients transferred to the ICU, we suggested that a majority of complications were diagnosed. Five, the centers included in the study used different methods to measure troponin and BNP/NT-pro-BNP. This heterogeneity of dosage could alter results on these biomarkers which should be interpreted with caution. Finally, we did not perform systematic evaluation of vascular age. This parameter could better precise cardiovascular risk of young individuals than age. However, vascular age provides a reflect of previous cumulation exposure to risk factors for arterial damage and subsequent cardiovascular risk. The report of clinical cardiovascular risk factors (age, sex, hypertension, dyslipidemia, diabetes, obesity, familial premature cardiovascular disease, smoking) enabled to have a clinical assessment of cardiovascular risk.

## 5. Conclusions

Through a multicenter observational study of patients with COVID-19 requiring admission to a hospital, we provide a comparison between 18–45-y-o patients and those > 45 y/o. Although the primary composite outcome of transfer to the ICU and in-hospital death occurred less frequently in young patients compared to older patients, COVID-19 seemed to have a particular CV impact in these patients with more myocarditis and pericarditis, less acute heart failure and a similar thrombotic complications rate in comparison with patients > 45 y/o.

## Figures and Tables

**Table 1:** Baseline characteristics of the study population stratified by age.

Variables	Patients > 45 y/o (*n* = 2547)	Patients 18–45 y/o (*n* = 321)	*p* Value	*n* Value
**Demographic Characteristics**				
Age (y/o)–median [IQR]	70.2 [60.0;81.4]	36.2 [30.8;40.8]	**<0.001**	2868
Male–*n* (%)	1488 (58.3)	174 (54.2)	0.790	2868
BMI–kg/m^2^	27.7 ± 6.0	28.9 ± 6.6	0.004	2489
≤25 kg/m^2^–*n* (%)	782 (35.4)	84 (30.2)	**0.001**	2489
25 to 30 kg/m^2^–*n* (%)	775 (35.1)	94 (33.8)		
30 to 35 kg/m^2^–*n* (%)	431 (19.5)	50 (18.0)		
>35 kg/m^2^–*n* (%)	223 (10.1)	50 (18.0)		
**Cardiovascular Risk Factors**				
Smoking–*n* (%)	340 (13.6)	36 (11.5)	0.337	2805
Hypertension–*n* (%)	1422 (56.1)	29 (9.2)	**<0.001**	2854
Diabetes–*n* (%)	656 (25.9)	20 (6.3)	**<0.001**	2855
Dyslipidemia–*n* (%)	783 (30.9)	15 (4.7)	**<0.001**	2854
Obesity *–*n* (%)	654 (29.6)	100 (36.0)	**0.034**	2489
Familial premature CVD–*n* (%)	42 (1.8)	15 (4.7)	0.226	2710
**Medical History**				
Any–*n* (%)	2048 (81.2)	124 (39.2)	**<0.001**	2838
COPD–*n* (%)	162 (6.4)	1 (0.3)	**<0.001**	2868
CKD ^†^–*n* (%)	392 (15.6)	11 (4.4)	**<0.001**	2831
Stroke–*n* (%)	248 (9.9)	3 (0.9)	**<0.001**	2832
PAD–*n* (%)	142 (5.7)	3 (0.9)	**0.001**	2833
Atrial fibrillation–*n* (%)	411 (16.3)	4 (1.3)	**<0.001**	2847
Heart failure–*n* (%)	241 (9.6)	5 (1.6)	**<0.001**	2823
CAD–*n* (%)	334 (13.1)	1 (0.3)	**<0.001**	2868
Malignancy–*n* (%)	401 (15.7)	13 (4.1)	**<0.001**	2868
VTE disease–*n* (%)	236 (9.2)	13 (4.1)	**0.001**	2868
Immunodeficiency–*n* (%)	126 (4.9)	21 (6.5)	0.273	2868
**Treatment before Hospitalization**				
None–*n* (%)	719 (28.2)	245 (76.3)	**<0.001**	2868
Anticoagulation–*n* (%)	408 (16.0)	9 (2.8)	**<0.001**	2868
Beta-blockers–*n* (%)	717 (28.1)	15 (4.7)	**<0.001**	2868
ACEi–*n* (%)	498 (19.5)	8 (2.5)	**<0.001**	2868
ARB–*n* (%)	460 (18.0)	8 (1.5)	**<0.001**	2868
Diuretics–*n* (%)	553 (21.7)	9 (2.8)	**<0.001**	2868
Statins–*n* (%)	645 (25.3)	7 (2.2)	**<0.001**	2868

Otherwise specified, data are presented as mean ± SD. Percentages are based on patients for whom data were available. * Obesity is defined as a BMI > 30 kg/m^2^. ^†^ CKD is defined as an eGFR ≤ 60 mL/min/1.73 m^2^. Abbreviations: ACEi, angiotensin converting enzyme inhibitor; ARBs, angiotensin receptor blocker; BMI, body mass index; CAD, coronary artery disease; CKD, chronic kidney disease; COPD, chronic obstructive pulmonary disease; CVD, cardiovascular disease; eGFR, estimated glomerular filtration rate; PAD, peripheral artery disease; VTE, venous thromboembolic; y/o, years old.

**Table 2:** Outcomes of the study population stratified by age.

Outcomes	Patients > 45 y/o (*n* = 2547)	Patients 18–45 y/o (*n* = 321)	*p* Value
Transfer to ICU or in-hospital death–*n* (%)	783 (30.7)	54 (16.8)	**<0.001**
In-hospital death–*n* (%)	358 (14.0)	3 (0.9)	<0.001 *
Transfer to ICU–*n* (%)	498 (19.6)	53 (16.5)	0.219
Length of stay–days	9.2 ± 5.8	6.4 ± 4.7	**<0.001**
Cardiovascular complications–*n* (%) Acute coronary syndrome	35 (1.4)	1 (0.3)	0.176 *
Ischemic stroke	21 (0.8)	1 (0.3)	0.502 *
Acute pulmonary embolism	90 (3.5)	16 (5.0)	0.251
Acute heart failure	183 (7.2)	3 (0.9)	<0.001 *
Acute pericarditis	12 (0.5)	7 (2.2)	0.003 *
Acute myocarditis	14 (0.6)	8 (2.5)	0.002 *

Otherwise specified, data are presented as mean ± standard deviation. Percentages are based on the total number in each column. * Fisher exact test. Abbreviations: ICU, intensive care unit; y/o, years old.

**Table 3:** Multivariate analysis for occurrence of primary outcome in young patients with COVID-19.

Variables	Odds Ratio	Confidence Interval 95%	*p* Value
Age	1.00	(0.94–1.05)	0.919
Male	2.53	(1.21–5.63)	**0.017**
BMI	1.07	(1.02–1.13)	**0.010**
Hypertension	2.33	(0.78–6.81)	0.121
Smoking	0.33	(0.07–1.20)	0.127
Previous cardiovascular complications *	4.19	(0.85–19.76)	0.068

Abbreviations: BMI, body mass index. Adjustment for center. * Previous cardiovascular complications correspond to the composites of history of heart failure, atrial fibrillation, peripheral artery disease, coronary artery disease or stroke prior to admission.

## Data Availability

Data will be provided on reasonable request.

## Supplementary Materials

The following are available online at https://www.mdpi.com/article/10.3390/jcdd8120165/s1, Supplementary Table S1. Anticoagulation regimen during hospitalization according to the age. Supplementary Table S2. Clinical, laboratory and radiologic findings on admission. Supplementary Table S3. Electrocardiographic and echocardiographic findings of patients according to the presence or absence of myocarditis. Supplementary Table S4. Electrocardiographic and echocardiographic findings of patients according to the presence or absence of pericarditis. Supplementary Table S5. Rates of cardiovascular complications stratified by the presence or absence of primary outcome during hospitalization in young patients. Supplementary Table S6. Outcomes of young patients according to sex. Supplementary Table S7. Sensitivity analysis of the study population divided by groups of ten years. Supplementary Table S8. Univariate analysis for occurrence of primary outcome in young patients with COVID-19. Supplementary Table S9. Subgroup analysis of the population without any comorbidities stratified by age. Supplementary Table S10. Outcomes in young patients according to troponin level. Supplementary Table S11. Outcomes in young patients according to natriuretic peptides level. Supplementary Table S12. Characteristics and outcomes of the patients transferred to the intensive care unit stratified by transfer timing. Supplementary Figure S1. Troponin level according to the age of the patients (logarithmic transformation). Supplementary Figure S2. Cardiovascular outcomes according to age. Supplementary Data 1. Critical COVID-19 France Investigators.
