# Supplementary material for: Cardiovascular Characteristics and Outcomes of Young Patients with COVID-19
Source: J Cardiovasc Dev Dis. 2021 Nov 26;8(12):165. doi: 10.3390/jcdd8120165 (PMC8704739; doi:10.3390/jcdd8120165)
Supplement: Supplementary file 1 [file jcdd-08-00165-s001.zip › jcdd-1416921-supplementary.pdf]

SUPPLEMENTAL MATERIAL

Supplementary Table S1. Anticoagulation regimen during hospitalization according to the age.

Anticoagulation regimen	>45 y/o (n = 2,385)	18-45 y/o (n = 300)
None	274 (11.5)	63 (21.0)
Prophylaxis dose	1362 (57.1)	183 (61.0)
Intermediate dose	238 (10.0)	33 (11.0)
Therapeutic dose	511 (21.4)	21 (7.0)

Data are presented as: absolute values (%).

The anticoagulation regimen was categorized into three groups: 1) prophylaxis dose (daily low molecular-weight heparin or twice daily subcutaneous unfractionated heparin); 2) intermediate dose (double the preventive dose); 3) therapeutic dose before hospitalization.

Supplementary Table S2. Clinical, laboratory and radiologic findings on admission.

Variables	Patients >45 y/o (n = 2,547)	Patients 18-45 y/o (n = 321)	*p* value	N value
**Clinical characteristics**				
Time from illness onset to hospitalization – days	6.7 ± 4.7	7.2 ± 4.3	0.065	2,772
NYHA class III or IV – n (%)	1,154 (52.1)	124 (44.6)	0.087	2,495
Heart rate – bpm	86 ± 18	93 ± 18	**<0.001**	2,621
Systolic pressure – mmHg	132 ± 22	125 ± 18	**<0.001**	2,825
Diastolic pressure – mmHg	74 ± 14	75 ± 13	0.257	2,825
Respiratory frequency – per min	23 ± 6	24 ± 7	0.516	2,110
Temperature – °C	37.5 ± 1.0	37.6 ± 1.0	0.072	2,825
O_2 saturation – %	94.6 ± 3.6	95.9 ± 3.4	**<0.001**	2,849
Glasgow score <15 – n (%)	184 (7.3)	9 (2.8)	**0.004**	2,834
HF signs – n (%)	182 (7.3)	7 (2.2)	**0.001**	2,819
SIC score ≥4 – n (%)	1,016 (68.6)	118 (62.1)	0.083	1,670
qSOFA ≥1 – n (%)	1,159 (62.0)	138 (58.0)	0.253	2,106
**Laboratory data**				
PaO_2 – mmHg	79.8 ± 26.7	90.0 ± 43.4	**0.001**	2,016
pH – IU	7.45 ± 0.06	7.45 ± 0.05	0.790	2,001
PaO_2/FiO_2 ratio <150 – n (%)	169 (9.7)	6 (2.9)	**0.002**	1,953
Lactate – mmol/L	1.46 ± 1.01	1.18 ± 0.57	**<0.001**	1,751
Leukocytes – g/L	7.35 ± 5.29	7.19 ± 3.66	0.484	2,822
Lymphocytes – g/L	1.28 ± 3.63	1.52 ± 1.60	**0.044**	2,780
Hemoglobin – g/dL	13.1 ± 2.0	13.5 ± 1.9	**<0.001**	2,830
C-Reactive Protein – mg/L	92.8 ± 77.4	70.7 ± 71.8	**<0.001**	2,754
Platelet – g/L	219 ± 100	231 ± 94	**0.032**	2,802
Prothrombin rate – %	84 ± 18	90 ± 12	**<0.001**	2,211
aPTT ratio – IU	1.2 ± 0.3	1.1 ± 0.1	**<0.001**	2,119
Creatinine – µmol/L	100.0 ± 95.0	81.7 ± 96.6	**0.002**	2,829
Aspartate aminotransferase – UI/L	55 ± 72	46 ± 36	**0.001**	2,603
Alanine aminotransferase – UI/L	45 ± 60	59 ± 150	0.130	2,609
Alkaline phosphatase – UI/L	92 ± 125	76 ± 50	**<0.001**	2,361
Gamma-glutamyl-transferase – UI/L	94 ± 136	76 ± 86	**0.003**	2,296
Albumin – g/L	31.4 ± 6.4	35.5 ± 6.4	**<0.001**	1,641
D-dimer – µg/L	1,707 ± 3801	1,223 ± 2180	**0.026**	1,153
Fibrinogen – g/L	6.1 ± 1.6	5.4 ± 1.9	**<0.001**	1,376
Elevated BNP or NT-pro-BNP* – n (%)	921 (57.0)	19 (11.9)	**<0.001**	1,774
Troponin elevation† – no (%)	542 (24.4)	28 (15.1)	**<0.001**	1,759
Positive SARS-CoV-2 RT-PCR – n (%)	2,373 (93.0)	293 (91.3)	0.317	2,873
**Abnormalities on chest CT**				
Severe parenchymal involvement (>50%) – n (%)	393 (19/7)	36 (14.4)	0.054	2,243

Otherwise specified, data are presented as mean ± SD. Percentages are based on patients for whom data were available.

* BNP >50 pg/mL or NT-pro-BNP >300 pg/mL

† Troponin above each center threshold

Abbreviations: aPTT, activated partial thromboplastin time; BNP, brain natriuretic peptide; CT, computed tomography; FiO2, inspired oxygen fraction; HF, heart failure; NT-pro-BNP, N-terminal pro-brain natriuretic peptide; NYHA, New York Heart Association; PaO_2, partial pressure of oxygen; qSOFA, quick sequential organ failure assessment score; RT-PCR, reverse-transcriptase polymerase chain reaction; SARS-CoV-2, severe acute respiratory syndrome coronavirus 2; SIC, sepsis-induced coagulopathy score; y/o, years old

Supplementary Table S3. Electrocardiographic and echocardiographic findings of patients according to the presence or absence of myocarditis.

Variables	All patients (n = 2,868)	Myocarditis		*p* value	N value
		No (n = 2,856)	Yes (n = 22)		
Age 18-45 y/o – n (%)	321 (11.2)	313 (11.0)	8 (36.4)	**0.002**	2,868
**Electrocardiographic characteristics**					
Heart rate – bpm	87 ± 18	87 ± 18	91 ± 25	0.409	2,624
Supraventricular arrhythmia – n (%)	260 (11.2)	259 (11.3)	1 (5.0)	0.718	2,312
Complete bundle branch block – n (%)	164 (7.7)	162 (7.7)	2 (10.0)	0.573	2,121
QT interval – msec	435 ± 51	435 ± 50	479 ± 77	**0.030**	1,162
**Echocardiographic characteristics**					
LVEF – n (%)<br>36-49%<br>≤35%	 50 (11.3)<br>53 (12.0)	 47 (11.0)<br>48 (11.2)	 3 (20.0)<br>5 (33.3)	**0.013**	443
LV dilatation – n (%)	48 (11.6)	43 (10.8)	5 (31.2)	**0.028**	413
LV hypertrophy – n (%)	100 (25.1)	97 (25.4)	3 (18.8)	0.770	398
RV dysfunction – n (%)	65 (16.1)	64 (16.4)	1 (7.7)	0.702	403
Pericardial effusion – n (%)	47 (11.3)	45 (11.1)	2 (15.4)	0.648	417

Otherwise specified, data are presented as mean ± SD.

Abbreviations: LVEF, left ventricle ejection fraction; LV, left ventricle; RV, right ventricle; y/o, years old

Supplementary Table S4. Electrocardiographic and echocardiographic findings of patients according to the presence or absence of pericarditis.

Variables	All patients (n = 2,868)	Pericarditis		*p* value	N value
		No (n = 2,859)	Yes (n = 19)		
Age 18-45 y/o – n (%)	321 (11.2)	314 (11.0)	7 (36.8)	**0.003**	2,868
**Electrocardiographic characteristics**					
Heart rate – bpm	87 ± 18	87 ± 18	83 ± 17	0.338	2,624
Supraventricular arrhythmia – n (%)	260 (11.2)	258 (11.2)	2 (11.8)	1.000	2,312
Complete bundle branch block – n (%)	164 (7.7)	164 (7.8)	0 (0.0)	0.210	2,121
QT interval – msec	435 ± 51	436 ± 51	417 ± 46	0.268	1,162
**Echocardiographic characteristics**					
LVEF – n (%)				0.332	443
36-49%	50 (11.3)	49 (11.4)	1 (8.3)		
<35%	53 (12.0)	50 (11.6)	3 (25.0)		
LV dilatation* – n (%)	48 (11.6)	46 (11.4)	2 (18.2)	0.460	413
LV hypertrophy† – n (%)	100 (25.1)	99 (25.4)	1 (11.1)	0.460	398
RV dysfunction‡ – n (%)	65 (16.1)	64 (16.3)	1 (10.0)	1.000	403
Pericardial effusion – n (%)	47 (11.3)	38 (9.4)	9 (81.8)	**<0.001**	417

Otherwise specified, data are presented as mean ± SD.

* LV dilatation was defined according to echocardiographic criteria: diastolic LV internal dimension assessed in M-mode tracing or 2D-guided linear measurements in parasternal long-axis view >58 mm in male or >52 mm in female, or a LV end-diastolic volume assessed via biplane disk summation method in apical four- and two-chamber views >150 mL or 74 mL/m^2 in male, or >106 mL or 61 mL/m^2 in female

† LV hypertrophy was defined as a LV mass index >115 g/m^2 in male or >95 g/m^2 in female measured in echocardiography

‡ RV dysfunction was defined by echocardiography as a TAPSE <17 mm and/or a pulsed Doppler S wave <9.5 cm/sec or a fractional area change <35%

Abbreviations: LVEF, left ventricle ejection fraction; LV, left ventricle; RV, right ventricle; y/o, years old

Supplementary Table S5. Rates of cardiovascular complications stratified by the presence or absence of primary outcome during hospitalization in young patients.

Outcomes	All patients 18-45 y/o (n = 321)	Patients 18-45 y/o without primary outcome (n = 267)	Patients 18-45 y/o with primary outcome (n = 54)
Acute coronary syndrome	1 (0.3)	0 (0.0)	1 (1.9)
Ischemic stroke	1 (0.3)	0 (0.0)	1 (1.9)
Acute pulmonary embolism	16 (5.0)	11 (4.1)	5 (9.3)
Acute heart failure	3 (0.93)	0 (0.0)	3 (5.6)
Acute myocarditis	8 (2.5)	6 (2.2)	2 (3.7)
Acute pericarditis	7 (2.2)	5 (1.9)	2 (3.7)

Data are presented as n (%).

Abbreviations: y/o, years old

Supplementary Table S6. Outcomes of young patients according to sex.

Outcomes	Patients 18-45 y/o (n = 321)		*p* value
	Women (n = 147)	Men (n = 174)	
Transfer to ICU or in-hospital death – n (%)	18 (12.2)	36 (20.7)	0 .062
In-hospital death – n (%)	1 (0.7)	2 (1.2)	1.000
Transfer to ICU – n (%)	18 (12.2)	35 (20.1)	0.082
Length of stay – days	5.8 ± 4.8	7.0 ± 4.7	**0.033**
Cardiovascular complications – n (%)			
Acute coronary syndrome	0 (0.0)	1 (0.3)	1.00
Ischemic stroke	1 (0.7)	0 (0.0)	0.458
Acute pulmonary embolism	2 (1.4)	14 (8.1)	**0.013**
Acute heart failure	1 (0.7)	2 (1.2)	1.000
Acute pericarditis	3 (2.0)	4 (2.3)	1.000
Acute myocarditis	3 (2.0)	5 (2.9)	0.731

Otherwise specified, data are presented as mean ± standard deviation. Percentages are based on the total number in each column.

Abbreviations: ICU, intensive care unit; y/o, years old

Supplementary Table S7. Sensitivity analysis of the study population divided by groups of ten years.

Variables	Patients 18-31 y/o (n = 83)	Patients 31-41 y/o (n = 161)	Patients 41-50 y/o (n = 268)	Patients >50 y/o (n = 2356)	*p* value	N value
**Demographic characteristics**						
Age – y/o	26.4 ± 3.5	36.4 ± 2.9	46.7 ± 2.8	72.4 ± 12.3	**<0.001**	2,868
Male – n (%)	41 (49.4)	87 (54.0)	169 (63.1)	1365 (57.9)	0.095	2,868
BMI – kg/m^2	27.9 ± 6.9	29.2 ± 6.8	29.2 ± 6.4	27.6 ± 5.9	**<0.001**	2,489
**Cardiovascular risk factors**						
Smoking – n (%)	12 (14.5)	14 (8.8)	26 (10.0)	324 (13.8)	0.091	2,805
Hypertension – n (%)	1 (1.2)	18 (11.4)	58 (21.8)	1374 (58.3)	**<0.001**	2,854
Diabetes – n (%)	2 (2.4)	12 (7.5)	27 (10.1)	635 (27.0)	**<0.001**	2,855
Dyslipidemia – n (%)	2 (2.4)	8 (5.0)	27 (10.2)	759 (32.4)	**<0.001**	2,854
Familial premature CVD – n (%)	0 (0.0)	0 (0.0)	3 (1.2)	41 (1.9)	0.236	2,710
**Medical history**						
COPD – n (%)	0 (0.0)	0 (0.0)	5 (1.9)	157 (6.7)	**<0.001**	2,868
CKD* – n (%)	0 (0.01)	8 (5/0)	11 (4.1)	282 (16.5)	**<0.001**	2,831
Stroke – n (%)	1 (1.2)	2 (1.3)	2 (0.8)	246 (10.6)	**<0.001**	2,832
PAD – n (%)	0 (0.0)	3 (1.9)	1 (0.4)	141 (6.1)	**<0.001**	2,833
Atrial fibrillation – n (%)	2 (2.4)	2 (1.3)	3 (1.1)	405 (17.4)	**<0.001**	2,847
Heart failure – n (%)	3 (3.7)	6 (3.8)	6 (2.3)	311 (13.4)	**<0.001**	2,823
CAD – n (%)	0 (0.0)	0 (0.0)	6 (2.2)	354 (15.0)	**<0.001**	2,868
Malignancy – n (%)	3 (3.6)	7 (4.4)	14 (4.8)	391 (16.6)	**<0.001**	2,868
VTE disease – n (%)	2 (2.4)	3 (1.9)	9 (3.4)	198 (8.4)	**<0.001**	2,868
**Treatment before hospitalization**						
Anticoagulation – n (%)	3 (3.6)	4 (2.5)	5 (1.9)	405 (17.2)	**<0.001**	2,868
Beta-blockers – n (%)	1 (1.2)	7 (4.4)	25 (9.3)	697 (29.6)	**<0.001**	2,868
ACEi – n (%)	1 (1.2)	5 (3.1)	18 (6.7)	482 (20.4)	**<0.001**	2,868
ARB – n (%)	1 (1.2)	5 (3.1)	19 (7.1)	443 (18.8)	**<0.001**	2,868
Diuretics – n (%)	1 (1.2)	4 (2.5)	14 (5.2)	541 (23.0)	**<0.001**	2,868
Statins – n (%)	1 (1.2)	4 (2.5)	21 (7.8)	626 (26.6)	**<0.001**	2,868
**Outcomes**						
Transfer to ICU or in-hospital death – n (%)	8 (9.6)	33 (20.5)	60 (22.4)	736 (31.2)	**<0.001**	2,868
In-hospital death – n (%)	0 (0.0)	1 (0.6)	7 (2.6)	353 (15.0)	**<0.001**	2,868
Transfer to ICU – n (%)	8 (9.6)	33 (20.5)	57 (21.3)	453 (19.2)	0.122	2,868
Length of stay – days	5.6 ± 4.3	6.9 ± 5.1	7.6 ± 5.2	9.3 ± 5.9	**<0.001**	2,868
Cardiovascular complications – n (%)<br>Acute coronary syndrome	0 (0.0)	0 (0.0)	1 (0.4)	35 (1.5)	0.234	2,868
Ischemic stroke	1 (1.2)	0 (0.0)	0 (0.0)	21 (0.9)	0.256	2,868
Acute pulmonary embolism	2 (2.4)	10 (6.2)	8 (3.0)	86 (3.7)	0.349	2,868
Acute heart failure	1 (1.2)	2 (1.2)	3 (1.1)	180 (7.6)	**<0.001**	2,868
Acute pericarditis	1 (1.2)	5 (3.1)	2 (0.8)	11 (0.5)	0.006	2,868
Acute myocarditis	2 (2.4)	6 (3.8)	0 (0.0)	14 (0.6)	**<0.001**	2,868

Otherwise specified, data are presented as mean ± SD. Percentages are based on patients for whom data were available.

* CKD is defined as an eGFR ≤ 60 mL/min/1.73m^2

Abbreviations: ACEi, angiotensin converting enzyme inhibitor; ARBs, angiotensin receptor blocker; BMI, body mass index; CAD, coronary artery disease; CKD, chronic kidney disease; COPD, chronic obstructive pulmonary disease; CVD, cardiovascular disease; eGFR, estimated glomerular filtration rate; ICU, intensive care unit; PAD, peripheral artery disease; VTE, venous thromboembolic; y/o, years old

Supplementary Table S8. Univariate analysis for occurrence of primary outcome in young patients with COVID-19.

Variables	Primary outcome		OR [CI 95%]	*p* value
	No (n=267)	Yes (n=54)		
**Demographic characteristics**				
Age (y/o) – median [IQR]	36.2 [30.3;40.9]	36.6 [33.3;40.6]	1.02 [0.98-1.06]	0.400
Male – n (%)	138 (51.7)	36 (66.7)	1.86 [1.01-3.51]	**0.045**
BMI – kg/m^2	28.5 ± 6.2	30.5 ± 7.7	1.04 [1.00-1.09]	0.052
**Cardiovascular risk factors**				
Smoking – n (%)	32 (12.4)	4 (7.4)	0.59 [0.16-1.57]	0.312
Hypertension – n (%)	19 (7.2)	10 (18.5)	2.92 [1.22-6.64]	**0.017**
Diabetes – n (%)	15 (5.6)	5 (9.3)	1.74 [0.53-4.77]	0.334
Dyslipidemia – n (%)	11 (4.2)	4 (7.4)	1.89 [0.49-5.86]	0.327
**Medical history**				
Any – n (%)	97 (37.0)	37 (50.0)	1.70 [0.94-3.08]	0.081
Stroke – n (%)	2 (0.8)	1 (1.9)	2.62 [0.08-32.9]	0.505
Atrial fibrillation – n (%)	2 (0.8)	2 (3.7)	5.02 [0.51-49.1]	0.151
Heart failure – n (%)	5 (1.9)	3 (5.6	3.10 [0.58-13.6]	0.168
Malignancy – n (%)	10 (3.8)	3 (5.6)	1.56 [0.32-5.42]	0.536
VTE – n (%)	7 (2.6)	2 (3.7)	1.50 [0.20-6.62]	0.644
**Treatment before hospitalization**				
None – n (%)	205 (76.8)	40 (74.1)	0.86 [0.45-1.74]	0.663
Anticoagulation – n (%)	8 (3.0)	1 (1.9)	0.69 [0.03-3.94]	0.722
Beta-blockers – n (%)	13 (4.9)	2 (3.7)	0.80 [0.11-3.05]	0.771
ACEi – n (%)	7 (2.6)	1 (1.9)	0.78 [0.03-4.67]	0.824
ARB – n (%)	6 (2.3)	2 (3.7)	1.75 [0.28-8.13]	0.536
Diuretics – n (%)	7 (2.6)	2 (3.7)	1.50 [0.20-6.62]	0.644
Statins – n (%)	4 (1.5)	3 (5.6)	3.89 [0.70-19.1]	0.113
Immunosuppressor – n (%)	17 (6.4)	4 (7.4)	1.12 [0.33-3.47]	0.751
**Clinical presentation**				
NYHA class III or IV – n (%)	91 (38.9)	33 (75.0)	4.65 [2.29-10.10]	**<0.001**
Heart rate – bpm	91 ± 17	106 ± 17	1.05 [1.03-1.07]	**<0.001**
Systolic arterial pressure – mmHg	124 ± 18	128 ± 18	1.01 [1.00-1.03]	0.174
Respiratory frequency – per min	23 ± 7	28 ± 8	1.09 [1.05-1.14]	**<0.001**
Temperature – °C	37.6 ± 1.0	38.1 ± 1.1	1.74 [1.30-2.33]	**<0.001**
FiO_2 – %	25 ± 8	35 ± 15	1.09 [1.06-1.13]	**<0.001**
SIC score ≥4 – n (%)	86 (55.8)	32 (88.9)	6.08 [2.26-21.70]	**<0.001**
qSOFA ≥1 – n (%)	100 (52.4)	38 (80.9)	3.78 [1.79-8.79]	**<0.001**
**Abnormalities on chest CT**				
Severe parenchymal involvement (>50%) – n (%)	18 (8.7)	18 (40.9)	7.13 [3.29-15.7]	**<0.001**

Otherwise specified, data are presented as mean ± SD. Percentages are based on patients for whom data were available.

Abbreviations: ACEi, angiotensin converting enzyme inhibitor; ARB angiotensin receptor blocker; BMI, body mass index; CT, computed tomography; FiO_2, inspired oxygen fraction; NYHA, New-York Heart Association; OR, odds ratio; qSOFA, quick sequential organ failure assessment score; SIC, sepsis-induced coagulopathy score; VTE, venous thromboembolism; y/o, years old

Supplementary Table S9. Subgroup analysis of the population without any comorbidities stratified by age.

Variables	Patients without any comorbidities		*p* value	N value
	>45 y/o (n = 473)	18-45 y/o (n = 192)		
**Demographic characteristics**				
Age – y/o	61.5 ± 11.5	34.8 ± 6.7	**<0.001**	665
Male – n (%)	279 (59.0)	107 (55.7%)	0.494	665
BMI – kg/m^2	27.2 ± 5.3	28.4 ± 6.5	**0.037**	563
**Clinical, biological and radiological data at admission**				
Time from illness onset to hospitalization – days	7.8 ± 4.4	7.4 ± 4.2	0.314	650
NYHA class III or IV – no (%)	213 (51.6)	69 (41.8)	**0.043**	578
Respiratory frequency – per min	24 ± 6	24 ± 8	0.793	492
Temperature – °C	37.3 ± 1.0	37.3 ± 1.0	0.812	658
O_2 saturation – %	95.1 ± 3.1	96.0 ± 3.5	**0.002**	662
HF signs – n (%)	9 (1.9)	3 (1.6)	1.000	656
SIC score ≥4 – n (%)	182 (66.2)	72 (64.3)	0.812	387
qSOFA ≥1 – n (%)	220 (63.4)	84 (58.7)	0.388	490
PaO_2 – mmHg	80 ± 22	90 ± 43	**0.009**	476
Lactate – mmol/L	1.2 ± 0.6	1.2 ± 0.6	0.353	402
Leukocytes – G/L	6.72 ± 2.90	6.93 ± 3.23	0.451	643
Lymphocytes – G/L	1.12 ± 0.89	1.56 ± 1.87	**0.003**	638
Haemoglobin – g/dL	13.6 ± 1.7	13.8 ± 1.8	0.442	646
Platelet – G/L	225 ± 97	219 ± 80	0.395	640
C-Reactive Protein – mg/L	95.2 ± 78.6	69.9 ± 60.7	**<0.001**	634
Prothrombin rate – %	90 ± 11	90 ± 12	0.637	519
aPTT ratio – IU	1.1 ± 0.2	1.1 ± 0.1	0.318	486
eGFR – mL/min/1.73m^2	96 ± 20	103 ± 18	**<0.001**	649
Aspartate aminotransferase –UI/L	62 ± 87	49 ± 40	**0.012**	595
Alanine aminotransferase – UI/L	52 ± 40	69 ± 190	0.249	595
Alkaline phosphatase – UI/L	80 ± 73	72 ± 43	0.092	527
Gamma-glutamyl-transferase – UI/L	88 ± 97	78 ± 72	0.197	510
Albumin – g/L	32.7 ± 6.1	35.3 ± 6.5	**0.001**	352
D-dimer – µg/L	1361 ± 2443	1177 ± 2101	0.517	292
Fibrinogen – g/L	6.2 ± 1.6	5.4 ± 1.8	**<0.001**	307
Elevated BNP or NT-pro-BNP* – n (%)	70 (28.1)	4 (4.2)	**<0.001**	344
Troponin elevation† – no (%)	63 (22.7)	17 (15.5)	0.145	387
Positive SARS-CoV-2 RT-PCR – n (%)	432 (92.3)	172 (90.1)	0.442	659
Severe parenchymal involvement (>50%) on chest CT – n (%)	84 (21.2)	24 (15.8)	0.195	549
**Outcomes**				
Transfer to ICU or in-hospital death – n. (%)	122 (25.8)	27 (14.1)	**0.001**	665
In-hospital death – n. (%)	19 (4.0)	1 (0.5)	**0.032**	665

Transfer to ICU – n. (%)	108 (22.8)	27 (14.1)	**0.015**	665
Length of stay – days	8.6 ± 6.0	6.3 ± 4.6	**<0.001**	665
Cardiovascular complication – n. (%)<br>Acute coronary syndrome	3 (0.6)	0 (0.0)	0.561	665
Ischemic stroke	2 (0.4)	0 (0.0)	1.000	665
Acute pulmonary embolism	25 (5.3)	9 (4.7)	0.902	665
Acute heart failure	7 (1.5)	1 (0.5)	0.449	665
Acute pericarditis	2 (0.4)	3 (1.6)	0.148	665
Acute myocarditis	1 (0.2)	5 (2.6)	**0.009**	665

Otherwise specified, data are presented as mean ± SD.

Abbreviations: aPTT, activated partial thromboplastin time; BNP, brain natriuretic peptide; BMI, body mass index; CT, computed tomography; eGFR, estimated glomerular filtration rate; HF, heart failure; NT-pro-BNP, N-terminal pro-brain natriuretic peptide; NYHA, New-York Heart Association; PaO_2, partial pressure of oxygen; qSOFA, quick sequential organ failure assessment score; RT-PCR, reverse-transcriptase polymerase chain reaction; SARS-CoV-2, severe acute respiratory syndrome coronavirus; SIC, sepsis-induced coagulopathy score; y/o, years old

Supplementary Table S10. Outcomes in young patients according to troponin level.

Outcomes	Patients 18-45 y/o with available troponin dosage (n = 185)		*p* value
	Troponin not elevated (n = 157)	Troponin elevated (n = 28)	
Transfer to ICU or in-hospital death – n (%)	27 (17.2)	10 (35.7)	**0.045**
In-hospital death – n (%)	1 (0.6)	1 (3.6)	0.280
Transfer to ICU – n (%)	26 (16.6)	10 (35.7)	**0.036**

Abbreviations: ICU, intensive care unit; y/o, years old

Supplementary Table S11. Outcomes in young patients according to natriuretic peptides level.

Outcomes	Patients 18-45 y/o with available BNP or NT-pro-BNP dosage (n = 159)		*p* value
	BNP or NT-pro-BNP not elevated (n = 140)	BNP or NT-pro-BNP elevated (n = 19)	
Transfer to ICU or in-hospital death – n (%)	29 (20.7)	3 (15.8)	0.767
In-hospital death – n (%)	2 (1.4)	1 (5.3)	0.319
Transfer to ICU – n (%)	28 (20.0)	3 (15.8)	1.000

Abbreviations: BNP, brain natriuretic peptide; ICU, intensive care unit; NT-pro-BNP, N-terminal pro-brain natriuretic peptide; y/o, years old

Supplementary Table S12. Characteristics and outcomes of the patients transferred to the intensive care unit stratified by transfer timing.

Variables	Transfer to the ICU <24 hours (n = 128)	Transfer to the ICU >24 hours (n = 421)	*p* value	N value
**Demographic characteristics**				
Age (y/o)	60.8 ± 13.7	62.8 ± 12.6	0.146	549
Male – n (%)	98 (76.6)	290 (68.9)	0.119	549
BMI – kg/m^2	29.3 ± 6.3	29.4 ± 6.1	0.871	510
**Cardiovascular risk factors – n (%)**				
Smoking	19 (15.2)	65 (15.9)	0.972	535
Hypertension	57 (45.2)	214 (51.2)	0.284	544
Diabetes	31 (24.2)	124 (29.6)	0.285	547
Dyslipidemia	37 (28.9)	129 (30.8)	0.768	547
Familial premature CVD	1 (0.9)	9 (2.3)	0.470	502
**Outcomes – n (%)**				
In-hospital death	19 (14.8)	55 (13.1)	0.712	549
Acute coronary syndrome	6 (1.4)	1 (0.8)	1.000	549
Ischemic stroke	8 (1.9)	1 (0.8)	0.692	549
Acute pulmonary embolism	34 (8.1)	8 (6.3)	0.624	549
Acute heart failure	36 (8.6)	7 (5.5)	0.343	549
Acute pericarditis	2 (0.5)	1 (0.8)	0.550	549
Acute myocarditis	6 (1.4)	1 (0.8)	1.000	549

Otherwise specified, data are presented as mean ± SD.

Abbreviations: BMI, body mass index; COVID-19, coronavirus disease 2019; CVD, cardiovascular disease; ICU, intensive care unit.

Supplementary Figure S1. Troponin level according to the age of the patients (logarithmic transformation).

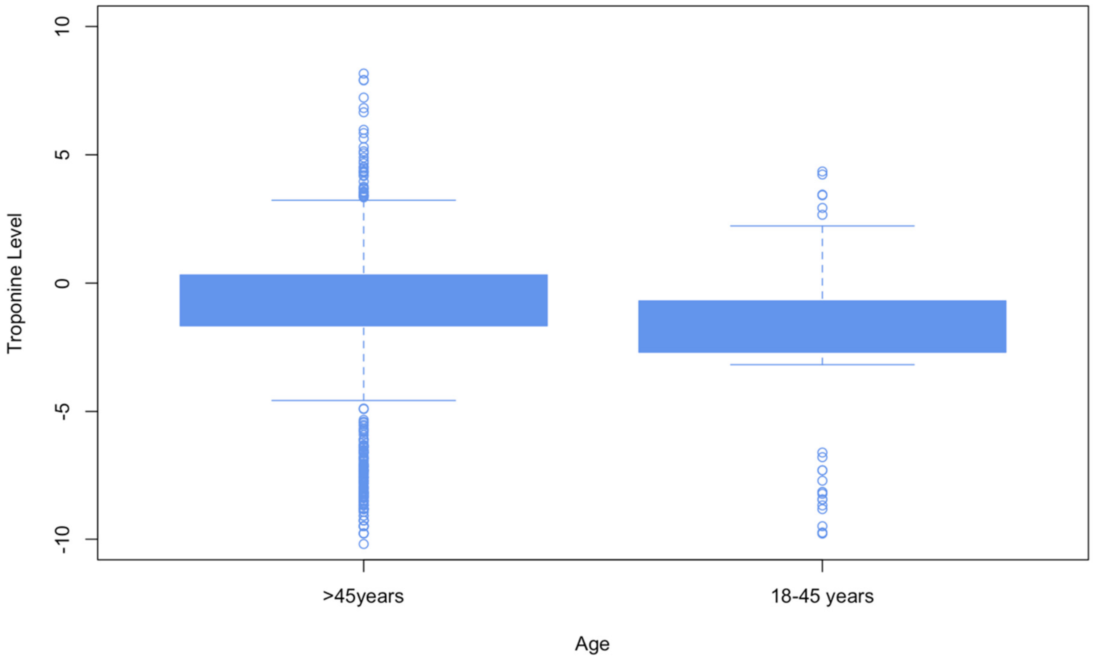

Supplementary Figure S2. Cardiovascular outcomes according to age.

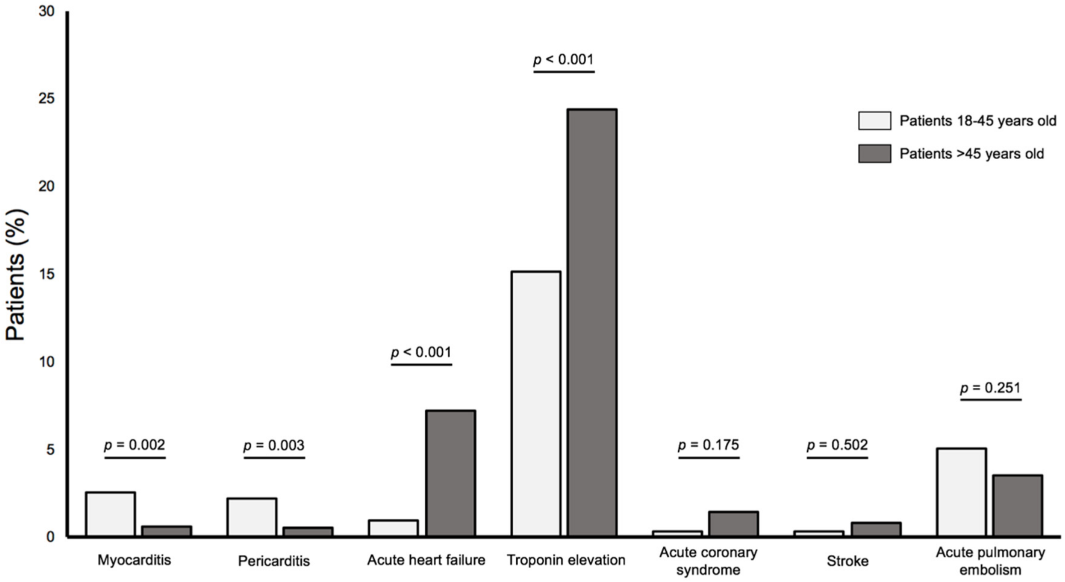

This figure shows the proportion of patients with each cardiovascular complication according to age (patients 18-45 years old and patients >45 years old). A *p* value is provided for each comparison.

Supplementary Data 1. Critical COVID-19 France Investigators.

ATTOU, Sabir, Centre Hospitalier Universitaire de Caen-Normandie, 14000 Caen, France

AUBRY, Matthieu, Hospices Civils de Lyon, Centre Hospitalier Universitaire, 69003 Lyon, France

BAGDADI, Imane, Institut Mutualiste Montsouris, 75014 Paris, France

BARBIN, Eva, Centre Hospitalier Régional de Orléans, 45100 Orléans, France

BARNAUD, Clément, Centre Hospitalier Régional de Orléans, 45100 Orléans, France

BENABOU, Léa, Centre Hospitalier Universitaire de Bordeaux, 33076 Bordeaux, France

BENMANSOUR, Othmane, Centre Hospitalier Régional de Orléans, 45100 Orléans, France

BONNET, Guillaume, Université de Paris, PARCC, INSERM, 75015 Paris, France

BOTHOREL, Léa, Centre Hospitalier Régional de Orléans, 45100 Orléans, France

BOUCHOT, Océane, Centre Hospitalier Annecy Genevois, 74370 Epagny Metz-Tessy, France

BOUFOULA, Ines, Centre Hospitalier Régional de Orléans, 45100 Orléans, France

CELLIER, Joffrey, Hôpital Européen Georges Pompidou, Université de Paris, 75015 Paris, France

CHABBI, Chaima, Centre Hospitalier Régional de Orléans, 45100 Orléans, France

CHAN, Camille, Centre Hospitalier Universitaire de Bordeaux, 33076 Bordeaux, France

CHAUMONT, Corentin, Hôpital Charles-Nicolle, Centre Hospitalier Universitaire de Rouen, 76000 Rouen, France

CHAVIGNIER, Diane, Centre Hospitalier Régional de Orléans, 45100 Orléans, France

CHEMALY, Pascale, Institut Cardiovasculaire Paris Sud, 91300 Massy, France

COHEN, Ariel, Saint Antoine Hospital, 75012 Paris, France

CUGNEY, Erwan, Centre Hospitalier Régional Universitaire de Nancy, 54511 Vandœuvre-Lès-Nancy, France

DARMON, Arthur, Hôpital Bichat-Claude-Bernard, APHP, Université de Paris, 75018 Paris, France

DELMOTTE, Thomas, Centre Hospitalier Universitaire de Reims, 51100 Reims, France

DELSARTE, Laura, Centre Hospitalier Régional Universitaire de Brest, 29200 Brest, France

DENEY, Antoine, Centre Hospitalier Universitaire de Toulouse, 31400 Toulouse, France

DOCQ, Clemence, Centre Hospitalier Universitaire de Lille, 59000 Lille, France

DOUAIR, Amine, Centre Hospitalier Annecy Genevois, 74370 Epagny Metz-Tessy, France

DUCEAU, Baptiste, Université de Paris, PARCC, INSERM, 75015 Paris, France

EZZOUHAIRI, Nacim, Centre Hospitalier Universitaire de Bordeaux, 33076 Bordeaux, France

FAUVEL, Charles, Centre Hospitalier Intercommunal Elbeuf, 76503 Saint-Aubin-lès-Elbeuf, France

FRAIX, Antoine, Centre Hospitalier Régional Universitaire de Nancy, 54511 Vandœuvre-Lès-Nancy, France

GAUTIER, Alexandre, Institut Cardiovasculaire Paris Sud, 91300 Massy, France

GENESTE, Laura, Centre Hospitalier Universitaire d'Amiens-Picardie, 80000 Amiens, France

GIORDANO, Gauthier, Centre Hospitalier Régional Universitaire de Nancy, 54511 Vandœuvre-Lès-Nancy, France

GODEAU, Guillaume, Institut Cardiovasculaire Paris Sud, 91300 Massy, France

GUILLEMINOT, Pierre, Centre Hospitalier Universitaire de Dijon, 21079 Dijon, France

KARSENTY, Clément, Centre Hospitalier Universitaire de Toulouse, 31400 Toulouse, France

LEBLON, Thiphaine, Centre Hospitalier Universitaire de Lille, Université Catholique de Lille, 59000 Lille, France

LEBOURDON, Romane, Centre Hospitalier Universitaire de Bordeaux, 33076 Bordeaux, France

LEVASSEUR, Thomas, Centre Hospitalier Intercommunal Fréjus-Saint-Raphaël, 83600 Fréjus, France

MA, Iris, Hôpital Européen Georges Pompidou, Université de Paris, 75015 Paris, France

MARSOU, Wassima, Centre Hospitalier Universitaire de Lille, Université Catholique de Lille, 59000 Lille, France

MASSIN, Michael, Centre Hospitalier Régional Universitaire de Nancy, 54511 Vandœuvre-Lès-Nancy, France

MECHERI, Yasmine, Centre Hospitalier Régional de Orléans, 45100 Orléans, France

MEVELEC, Marine, Centre Hospitalier Régional de Orléans, 45101 Orléans, France

MIKA, Delphine, Université Paris-Saclay, Inserm, UMR-S 1180, 92296 Chatenay-Malabry, France

NOIRCLERC, Nathalie, Centre Hospitalier Annecy Genevois, 74370 Epagny Petz-Tessy, France

PACE, Nathalie, Centre Hospitalier Régional Universitaire de Nancy, 54511 Vandœuvre-Lès-Nancy, France

PANAGIDES, Vassili, Centre Hospitalier Universitaire de Marseille, 13005 Marseille, France

PASTIER, Julie, Centre Hospitalier Universitaire de Dijon, 21079 Dijon, France

PERIN, Benjamin, Centre Hospitalier Régional Universitaire de Nancy, 54511 Vandœuvre-Lès-Nancy, France

PEZEL, Théo, Cardiology, Lariboisière hospital, APHP, University of Paris, 75010 Paris, France

POMMIER, Thibaut, Centre Hospitalier Universitaire de Dijon, 21079 Dijon, France

RIBEYROLLES, Sophie, Institut Mutualiste Montsouris, 75014 Paris, France

SAGNARD, Audrey, Centre Hospitalier Universitaire de Dijon, 21079 Dijon, France

SUTTER, Willy, Université de Paris, PARCC, INSERM, 75015 Paris, France

TRIMAILLE, Antonin, Nouvel Hôpital Civil, Centre Hospitalier Régional Universitaire de Strasbourg, 67000 Strasbourg, France

WALDMANN, Victor, Université de Paris, PARCC, INSERM, 75015 Paris, France

WEIZMAN, Orianne, Centre Hospitalier Régional Universitaire de Nancy, 54511 Vandœuvre-Lès-Nancy, France

YVOREL, Cédric, Centre Hospitalier Universitaire de Saint-Etienne, 42270 Saint-Priest-en-Jarez

ZAKINE, Cyril, Clinique Saint-Gatien, 37540 Saint-Cyr-sur-Loire, France

Supplementary Data 2. STROBE Statement.

	Item	Recommendation	Page
**Title and abstract**	1	(*a*) Indicate the study's design with a commonly used term in the title or the abstract	**1**
		(*b*) Provide in the abstract an informative and balanced summary of what was done and what was found	**1**
		**Introduction**	
Background/rationale	2	Explain the scientific background and rationale for the investigation being reported	**2**
Objectives	3	State specific objectives, including any prespecified hypotheses	**2**
		**Methods**	
Study design	4	Present key elements of study design early in the paper	**2-3**
Setting	5	Describe the setting, locations, and relevant dates, including periods of recruitment, exposure, follow-up, and data collection	**2-3**
Participants	6	(*a*) Give the eligibility criteria, and the sources and methods of selection of participants. Describe methods of follow-up	**2**
		(*b*) For matched studies, give matching criteria and number of exposed and unexposed	**NA**
Variables	7	Clearly define all outcomes, exposures, predictors, potential confounders, and effect modifiers. Give diagnostic criteria, if applicable	**3**
Data sources/ measurement	8*	For each variable of interest, give sources of data and details of methods of assessment (measurement). Describe comparability of assessment methods if there is more than one group	**3**
Bias	9	Describe any efforts to address potential sources of bias	**3**
Study size	10	Explain how the study size was arrived at	**3**
Quantitative variables	11	Explain how quantitative variables were handled in the analyses. If applicable, describe which groupings were chosen and why	**3**
Statistical methods	12	(*a*) Describe all statistical methods, including those used to control for confounding	**3**
		(*b*) Describe any methods used to examine subgroups and interactions	**3**
		(*c*) Explain how missing data were addressed	**NA**
		(*d*) If applicable, explain how loss to follow-up was addressed	**NA**
		(*e*) Describe any sensitivity analyses	**NA**
		**Results**	
Participants	13*	(a) Report numbers of individuals at each stage of study—e.g. numbers potentially eligible, examined for eligibility, confirmed eligible, included in the study, completing follow-up, and analysed	**3-4**
		(b) Give reasons for non-participation at each stage	**3-4**
		(c) Consider use of a flow diagram	**Figure 1**
Descriptive data	14*	(a) Give characteristics of study participants (eg demographic, clinical, social) and information on exposures and potential confounders	**4**
		(b) Indicate number of participants with missing data for each variable of interest	**Table 1**
Outcome data	15*	Report numbers of outcome events or summary measures over time	**4-5**<br>**Table 3**

Main results	16	(*a*) Give unadjusted estimates and, if applicable, confounder-adjusted estimates and their precision (eg, 95% confidence interval). Make clear which confounders were adjusted for and why they were included	**4-5**
		(*b*) Report category boundaries when continuous variables were categorized	**4-5**
		(*c*) If relevant, consider translating estimates of relative risk into absolute risk for a meaningful time period	**NA**
Other analyses	17	Report other analyses done—eg analyses of subgroups and interactions, and sensitivity analyses	**94-5**
**Discussion**			
Key results	18	Summarise key results with reference to study objectives	**6**
Limitations	19	Discuss limitations of the study, taking into account sources of potential bias or imprecision. Discuss both direction and magnitude of any potential bias	**8**
Interpretation	20	Give a cautious overall interpretation of results considering objectives, limitations, multiplicity of analyses, results from similar studies, and other relevant evidence	**6-8**
Generalisability	21	Discuss the generalisability (external validity) of the study results	**6-8**
**Other information**			
Funding	22	Give the source of funding and the role of the funders for the present study and, if applicable, for the original study on which the present article is based	**9**
